# Midday meals do not impair mouse memory

**DOI:** 10.1038/s41598-018-35427-y

**Published:** 2018-11-19

**Authors:** Sarah C. Power, Mateusz J. Michalik, Sylvie Couture-Nowak, Brianne A. Kent, Ralph E. Mistlberger

**Affiliations:** 1 Department of Psychology, Simon Fraser University, Burnaby, Canada; 2 Djavad Mowafaghian Centre for Brain Health, Department of Medicine, Division of Neurology, University of British Columbia, Vancouver, Canada

## Abstract

Nocturnal mice fed in the middle of the light period exhibit food anticipatory rhythms of behavior and physiology under control of food-entrainable circadian clocks in the brain and body. This is presumed to be adaptive by aligning behavior and physiology with predictable mealtimes. This assumption is challenged by a report that daytime feeding schedules impair cognitive processes important for survival, including object memory and contextual fear conditioning assessed at two times of day. To further evaluate these effects, mice were restricted to a 6 h daily meal in the middle of the light or dark period and object memory was tested at four times of day. Object memory was not impaired by daytime feeding, and did not exhibit circadian variation in either group. To determine whether impairment might depend on methodology, experimental procedures used previously to detect impairment were followed. Daytime feeding induced food anticipatory rhythms and shifted hippocampal clock genes, but again did not impair object memory. Spontaneous alternation and contextual fear conditioning were also not impaired. Hippocampal memory function appears more robust to time of day and daytime feeding schedules than previously reported; day-fed mice can remember what they have seen, where they have been, and where it is dangerous.

## Introduction

Nocturnal rats and mice with free access to food are active and eat predominantly at night, under control of a light-dark (LD)-entrainable circadian pacemaker in the suprachiasmatic nucleus (SCN). If food availability is restricted to the middle of the light period for a week or more, a bout of activity emerges in anticipation of the daily meal^1–3^, and circadian clocks in most peripheral organs and tissues shift to align with mealtime^4,5^. These circadian adjustments to daily feeding schedules do not involve the SCN pacemaker, as the SCN, when entrained to LD cycles, is not shifted by daytime feeding^6–8^, and SCN ablation does not disrupt food anticipatory rhythms or prevent entrainment of peripheral clocks to scheduled feeding^9–11^.

Food-entrainable circadian timing has been viewed as an adaptation that ensures appropriate alignment of foraging behavior and physiology with feeding opportunities, even when these opportunities are limited to times of day that are outside of the organism’s preferred temporal niche. This adaptive view is challenged by a report from Loh and colleagues^12^ that daytime restricted feeding schedules markedly impair performance on two widely used tests of hippocampus-dependent memory function. In that study, C57BL6 mice were entrained to a standard 12 h:12 h LD cycle and then restricted to a 6 h feeding opportunity in the middle of the dark period (night-fed) or the middle of the light period (day-fed) for two weeks. Object memory was then tested either in the day or the night using the Novel Object Recognition (NOR) test. This test is based on the natural preference of mice for novel objects over familiar objects^13^. When provided with a choice between two such objects, preference for the novel object implies memory of the previously encountered object. Time spent exploring the two objects can be expressed as a discrimination ratio and used as a metric of object memory. Loh *et al*.^12^ found that night-fed mice exhibited a daily rhythm in performance, with significant preference for the novel object when tested at night (typical active phase) but no preference when tested in the day (typical rest phase). By contrast, day-fed mice failed to show a significant preference for the novel object at either test time. Contextual fear conditioning was similarly impaired in day-fed mice tested in the day or the night. The study also confirmed that circadian rhythms of hippocampal electrophysiology and clock gene expression are markedly shifted in day-fed mice, whereas clock gene rhythms in the SCN pacemaker remain aligned with the LD cycle^14–17^. The results were interpreted as evidence that shifting of hippocampal rhythms relative to the SCN pacemaker represents a state of circadian misalignment that disrupts hippocampal memory functions.

To our knowledge, this is the first report that circadian adjustments to a daily feeding schedule can impair brain functions essential for survival in natural environments. Daily rhythms of hippocampus-dependent memory performance have been reported previously^18–21^. It is possible that daytime restricted feeding shifts these rhythms, and that memory functions may not be impaired at specific circadian phases, such as prior to mealtime, when day-fed mice are spontaneously awake and actively seeking food. This might be missed if only two daily time points are sampled, as in the Loh *et al*.^12^ study. To test this hypothesis, we compared performance of day-fed and night-fed mice on the NOR test at 4 times of day, with additional procedural modifications and a larger sample size. We confirmed a shift of circadian clock genes in the hippocampus in day-fed mice, but observed no impairment of object memory in either group at any of the four test times. To determine whether deficits in object memory might depend on specific methodological details, the procedures and apparatus used by Loh *et al*.^12^ were replicated as closely as possible. Again, day-fed and night-fed groups performed well at all test times. There was also no impairment of spontaneous alternation or contextual fear conditioning, two additional tests sensitive to hippocampal disruption^22,23^. These results support the view that stable alterations in circadian timing induced by daily feeding schedules represent an adaptive re-alignment of internal temporal order, rather than a ‘misalignment’ that disrupts cognitive functions important for survival.

## Methods

### Animals and Housing

Young male C57BL/6 mice (N = 110, 2–4 months age) were obtained from Charles River (QC, Canada). Separate cohorts were used to test NOR in a Y-maze (Experiment 1, N = 34) and in an open field (Experiment 2, N = 37). A third cohort (N = 40) was used to test spontaneous alternation (Experiment 3a) and contextual fear conditioning (Experiment 3b). All mice were single-housed in a 12:12 LD cycle (white LED, ~15 lux at cage bottom) with food and water available *ad libitum* for at least two weeks prior to restricted feeding. Room temperature was maintained at ~22 °C. In Experiment 1, locomotor activity was recorded continuously using Clocklab data acquisition and analysis software (Actimetrics, USA). All procedures were approved by the Simon Fraser University Animal Care Committee (Protocol 1208P-16) and all experiments were performed in accordance with relevant guidelines and regulations.

### Restricted Feeding

Mice in Experiment 1 (NOR Y-maze test) were maintained on a restricted feeding schedule for at least 40 days prior to testing to ensure stable entrainment to the feeding schedule. The schedule was initiated with an 18 h food deprivation starting at Zeitgeber Time (ZT) 12 (where ZT0 is lights-on, by convention). Food was then made available each day at ZT3 (3 h after lights-on; day-fed) or ZT15 (3 h after lights-off; night-fed). Meal duration was 10 h on the first day, reduced by 1 h/day for the next four days, and maintained at 6 h (ZT3–9 or ZT15-21) through the end of NOR testing. Food consumed and body weights were measured daily for the first 2 weeks.

Mice in Experiment 2 (NOR open field test) and Experiment 3 (spontaneous alternation and contextual fear conditioning tests) were maintained on the restricted feeding schedule as described by Loh *et al*.^12^. Accordingly, the mice were food deprived for 24 h and then provided food for 6 h/day from ZT3-9 or ZT15-21 for 2 weeks, with test procedures beginning on day 15 of restricted feeding.

Experiment timelines and apparatus for each experiment are illustrated in Supplementary Figure S1.

### Experiment 1: NOR test in Y-maze apparatus

The Y-maze has advantages over the open field for tests of object memory. The narrow arms of the Y-maze reduce anxiety and therefore encourage exploration. The high walls limit the use of spatial cues, encouraging attention to the objects. The Y-maze was made of homogenous opaque white Perspex (described in^24^). Walls were 30 cm high and each arm was 16 cm in length and 8 cm wide. A digital video camera was mounted above the maze to record all trials. One arm was used as the start arm, and the other two arms were used to present the objects secured to the maze floor using Blu-tack ^TM^. The maze and objects were wiped with a 50% ethanol solution and dried between trials. The objects used and side of the maze in which the novel object was presented were counterbalanced. All tests in the Y-maze were performed under dim light (white incandescent, ~10 lux).

Mice were habituated to gentle handling by 3–4 min handling sessions repeated daily for ~2 weeks prior to NOR testing. Mice were habituated to the Y-maze by being placed in the apparatus for 10 min on each of the two days immediately prior to the first NOR test. Each NOR test consisted of two familiarization trials and one choice trial, scheduled at 24 h intervals over successive days. During familiarization trials, mice were placed in one end of the Y-maze with two identical objects at the other two ends and allowed to explore for 5 min. During the choice trial, the mice were placed in the Y-maze and presented with one *familiar* object, which was identical to the objects presented during the familiarization phase, and one *novel* object, which the mice had not encountered previously. The mice were given 5 min to explore the maze and objects. The mice were then returned to their home cages and maintained on the restricted feeding schedules for another 4 days. The 3-day test sequence was then repeated at another time of day, and the complete 7-day sequence was repeated until each mouse had been tested at all four time points (ZT3, 9, 15 and 21, counterbalanced for order).

Exploratory activity levels are affected by hunger. Therefore, during the 24 h prior to each choice trial, the mice were provided a small meal (25% of average daily food intake) every 6 h (ZT3, 9, 15 and 21), in place of one large meal at ZT3 or ZT15. This ensured that time since lasting feeding was equivalent on each choice trial.

### Experiment 2: NOR test in open field apparatus

The procedures used in Experiment 2 were intended to match those of Loh *et al*.^12^ as closely as possible. Accordingly, testing was conducted in opaque white boxes (55 × 37 × 33 cm). The objects (from Experiment 1) were placed centrally, equidistant from each other and the sides of the open field. Testing at ZT9 was conducted in the light (~30 lux) and testing at ZT21 under dim red light (<2 lux). The mice were first habituated to the open field by allowing them to explore the field without objects for 10 min on the two days prior to the first familiarization trial. The mice then received two 10 min familiarization trials and a 5 min choice trial, at 24 h intervals, at either ZT9 or ZT21. Between trials, the apparatus and objects were wiped clean with 10% Windex. After completion of NOR testing, the mice were euthanized for analysis of clock gene expression in the hippocampus.

### NOR data analysis

Object exploration was scored when a mouse directed its nose toward an object at a distance of 2 cm or less. This included both sniffing and touching the object while looking at it. Climbing, sitting, or chewing on the object were not scored as exploration. Scoring was done by an experienced coder (BAK) blind to the conditions, using JWatcher (v1.0, JWatcher, USA). The program assigns separate keys to each object. Exploration of each object was recorded by pressing the appropriate key at the onset and end of a bout. Mice that explored for <3 sec during the familiarization or choice phase were excluded from the analysis. Preference for the novel object was quantified by a Discrimination Index (DI), where *DI* = *time exploring novel object/(time exploring novel object* + *time exploring familiar object)*. DI scores >0.5 represent a novelty preference.

### Hippocampus Extraction and Quantitative Polymerase Chain Reaction

Circadian rhythms are driven by autoregulatory transcription-translation feedback loops involving a group of clock genes that includes *Bmal1* and *Per2*^25^. Clock genes are responsible for circadian oscillations at the cellular level and expression can be used to assess tissue-specific circadian phase. On the day after the NOR testing and the last scheduled meal, day-fed and night-fed mice in Experiment 2 were euthanized via CO_2_ at ZT3, 6, 9 or 15, without feeding on that day. The mice were decapitated and brains were rapidly extracted and cooled in ice-cold Hank’s balanced salt solution (HBSS; Millipore-Sigma H1641, St Louis, MO) supplemented with Hepes buffer (Millipore-Sigma H0887, St Louis, MO) and NaCHO_3_ (Millipore-Sigma S8761, St Louis, MO). Brains were maintained in 4 °C HBSS and 1100-micron sections beginning at ~1.58 mm posterior to bregma^26^ were sectioned on a vibratome. Dorsal hippocampus was separated using a scalpel and immediately placed in a 2 mL Eppendorf tube and flash frozen on dry ice. Tissue was kept at −80 °C until RNA isolation. RNA was isolated using Trizol reagent (Thermo Fisher Scientific, 15596018, Waltham, MA) according to manufacturer’s instructions and concentrations were quantified using a spectrometer. RNA (500 ng) was reverse-transcribed to cDNA using High-Capacity cDNA Reverse Transcription Kit (Thermo Fisher Scientific, 4368814, Waltham, MA). Quantitative polymerase chain reaction (qPCR) of *Per2* was performed using 2 μL of cDNA with SYBR Green FastMix (Quanta Biosciences, 95073, Gaithersburg, MD) in a StepOnePlus real time PCR system (Thermo Fisher Scientific, Waltham, MA). mRNA expression of *Per2* and *Bmal1* was normalized to *Rplp0*. Primers used are provided in Supplementary Table S1.

### Experiment 3a. Spontaneous alternation

Although often referenced as a test of hippocampal memory function, object recognition assayed by the methods described in Loh *et al*.^12^, and used in Experiments 1 and 2 here, is understood to be dependent on the perirhinal cortex rather than the hippocampus^25,26^. Therefore, a third cohort of day-fed and night-fed mice was tested for spontaneous alternation and contextual fear conditioning, two consensus tests of hippocampal function^22,23^. The mice were habituated to handling and maintained on a 6 h daytime or nighttime meal for 14 days, as in Experiment 2. On day 15 of restricted feeding, spontaneous alternation was tested in separate groups at ZT2 and ZT14 (N = 10 day-fed and night-fed at each time) using the Y-maze described in Experiment 1. The mice were naïve to the apparatus. During the test the mice were confined to one arm of the Y-maze for 1 min. The mice were then allowed 7 min to explore all three arms of the maze. An alternation was judged to have occurred after a mouse visited three different arms of the maze on three successive arm visits. For example, the sequence ABBACCABACBA contains 5 alternations (BAC, CAB, BAC, ACB, CBA). Total alternations were then divided by total number of alternation opportunities (total arm visits minus one) to yield an alternation percent score.

### Experiment 3b. Contextual fear conditioning

Two days after the spontaneous alternation test, on days 17 and 18 of restricted feeding, contextual fear conditioning was assessed using an operant conditioning chamber equipped with a conducting floor (29.53 × 23.5 × 20.96 cm; Med Associates VFC-008-LP, Fairfax, VT). Day-fed and night-fed mice were placed in the chamber (conditioned stimulus) at either ZT2 or ZT14 (as in Loh *et al*.^12^) and allowed to freely explore for 3 min. Following this familiarization period, the mice received a mild 2 sec foot shock with a 0.2 mA current (unconditioned stimulus) delivered through the floor bars. The foot shock was repeated 64 sec later. After a final 64 sec interval, the mice were returned to their home cage. The mice were placed in the shock chamber 24 h later and recorded for 6 min to assess contextual freezing. Freezing was defined as immobility and assessed by an experienced coder blind to experimental conditions. Observations were taken every 8 sec during the 64 sec interval immediately prior to the first foot shock (8 observations) and during the entire 6 min test phase the next day (45 observations). Data were analyzed as a percent freezing score (percentage of observations scored as freezing).

### Statistics

NOR DI scores, alternation percent, freezing percent and qPCR data were statistically evaluated using mix-model or ordinary two-way-ANOVAs, or one-way ANOVA, as appropriate (Prism 6, Graphpad Software Inc,. La Jolla, USA).

## Results

### Experiment 1. Daytime feeding does not impair NOR in a Y-maze

**Figure 1:**
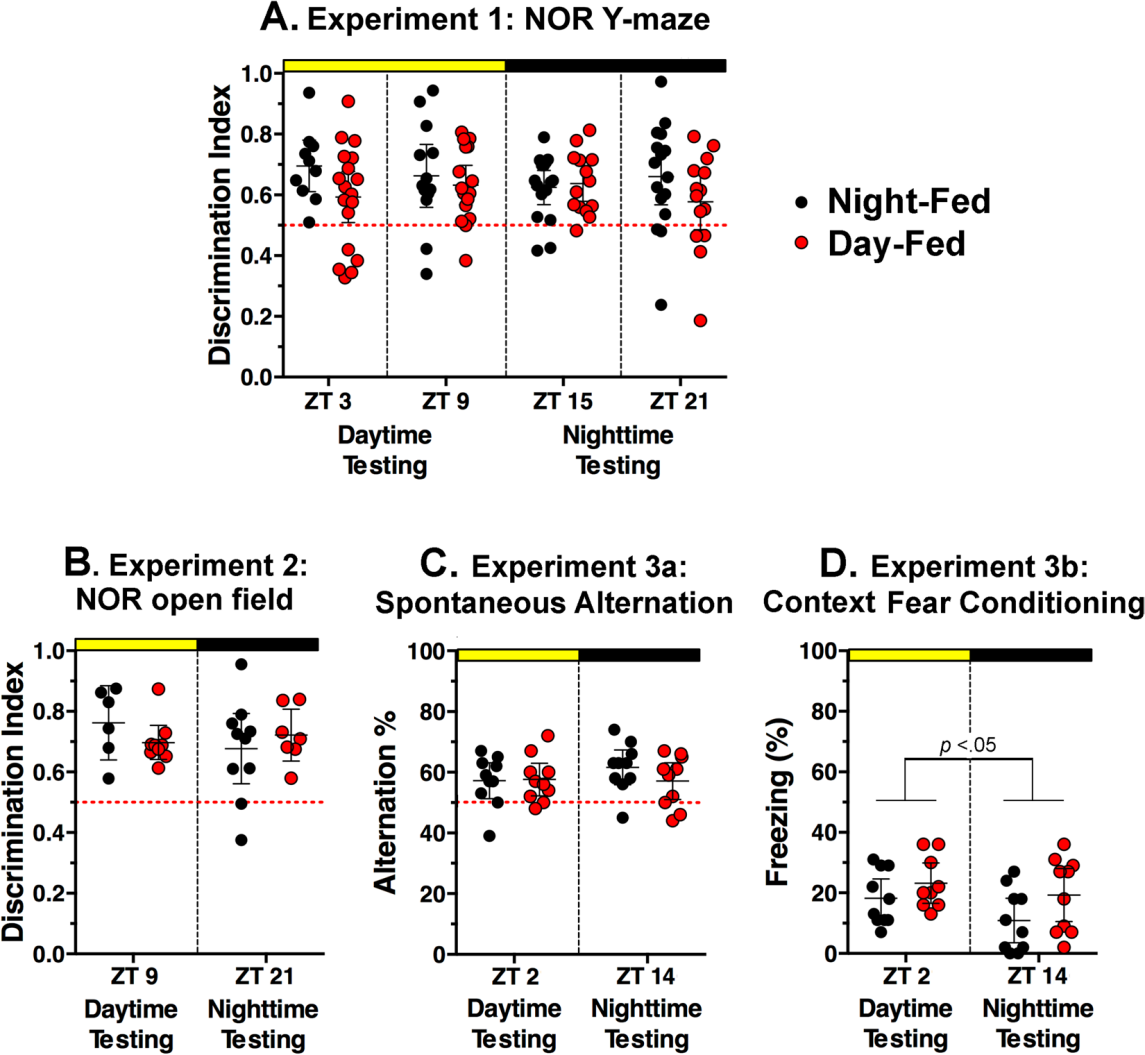
Effects of feeding schedules and time of day on test performance. (**A**) Experiment 1. Discrimination Index (DI, time spent exploring the novel object as a percentage of time spent exploring both objects) scores on novel object recognition (NOR) test in a Y-maze. Chance level is 0.5 (red dotted line). (**B**) Experiment 2. DI scores on NOR test in an open field. (**C**) Experiment 3a. Alternation percentages in a Y-maze. Chance level is 50% (red dotted line). (**D**) Experiment 3b. Freezing % in shock box, 24 h after receiving foot shock.

To assess whether meal timing affects object memory, NOR was tested at 4 times of day in a Y-maze. The percentage of trials on which mice met the 3-sec minimum object exploration criterion did not differ between the day-fed (62/72) and night-fed (55/64) groups (*z* = 0.029, *p* = 0.97). The majority of mice in both day-fed and night-fed groups exhibited a discrimination index >0.5 at each time point, indicating memory of the previously encountered object (Fig. 1A; Supplementary Fig. S2). A two-way repeated measures ANOVA of discrimination index scores from mice that met criterion on all trials confirmed no significant effect of time of day (*F*_3,63_ = 0.24, *p* = 0.87) or feeding schedule (*F*_1,21_ = 3.46, *p* = 0.08) and no significant interaction (*F*_3,63_ = 0.98, *p* = 0.40). Total object exploration (novel plus familiar) also did not exhibit a significant effect of time of day (*F*_3,63_ = 1.86, *p* = 0.14), feeding schedule (*F*_1,21_ = 0.12, *p* = 0.73) or interaction (*F*_3,63_ = 2.37, *p* = 0.08) (Supplementary Fig. S3).

### Experiment 2. Daytime feeding does not impair NOR in an open field

To identify potential procedural variables that might explain the discrepancy between the results of Experiment 1 and those reported previously, a replication experiment was attempted after consulting with the authors of the original study^12^. Accordingly, the feeding schedules were maintained for 14 days rather than >40 days and NOR was assessed in an open field apparatus, in separate groups tested at ZT9 or ZT21. The percentage of trials on which mice met the 3-sec minimum object exploration criterion did not differ between the day-fed (15/20) and night-fed (18/20) groups (*z* = 1.25 *p* = 0.21). Again, the majority of mice in the day-fed and night-fed groups exhibited a discrimination index >0.5, indicating memory of the previously encountered object, at both time points (Fig. 1B). There was no significant effect of time of day (*F*_1,28_ = 0.49, *p* = 0.48) or feeding schedule (*F*_1,28_ = 0.05, *p* = 0.82), and no significant interaction (*F*_1,28_ = 1.62, *p* = 0.21). Total object exploration also exhibited no effect of time of day (*F*_1,28_ = 2.21, *p* = 0.14), feeding schedule (*F*_1,28_ = 0.70, *p* = 0.41), or interaction (*F*_1,28_ = 1.06, *p* = 0.31) (Supplementary Fig. S3).

### Experiment 2. Restricted feeding schedules induce robust food anticipatory activity rhythms

**Figure 2:**
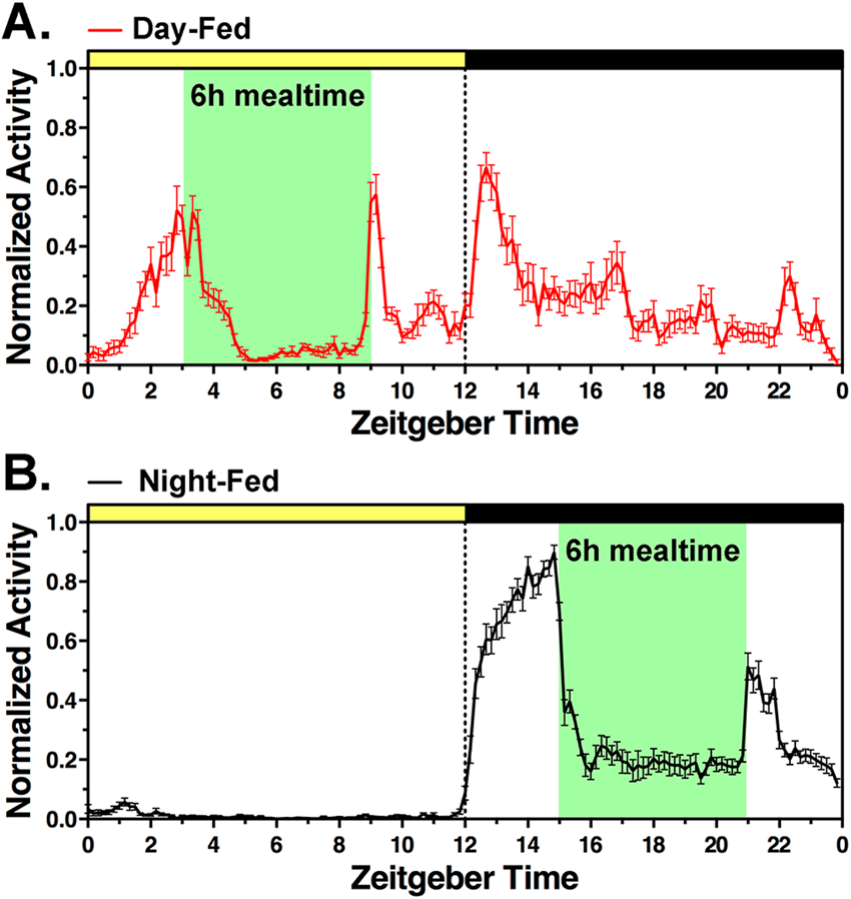
Effects of feeding schedules and time of day on locomotor activity in (**A**) day-fed and (**B**) night-fed mice in Experiment 1. Activity data for individual mice were first normalized and then averaged across the last 5 days of restricted feeding prior to habituation sessions. Day- and night-tested subgroups were pooled. Group means are plotted ± SEM. Mealtimes are denoted by green shading.

Daytime or nighttime restricted feeding schedules induce a daily rhythm of food anticipatory activity. To confirm that the 14-day restricted feeding schedules employed here were effective in inducing food anticipation, home cage locomotor activity was monitored continuously using infrared motion sensors. As expected, all mice in the day-fed and the night-fed groups exhibited a prominent bout of activity beginning 1–3 h prior to scheduled feeding each day (Fig. 2).

A two-way repeated measures ANOVA on food intake measured daily for the first 14 days of restricted feeding prior to NOR testing revealed significant effects of restricted feeding day (*F*_*1*3,*455*_ = 28.8, *p* < 0.0001) and mealtime (*F*_*1*,*35*_ = 22.6; *p* < 0.001), and a significant interaction (*F*_*13*,*455*_ = 9.21; *p* < 0.001). Day-fed mice ate less than night-fed mice on days 1, 3 and 4 of restricted feeding (Sidak’s multiple comparisons test, p < 0.05), but food intake did not differ between groups thereafter (Supplementary Fig. S4A). A two-way repeated measures ANOVA on body weights measured daily for the first 14 days of restricted feeding revealed a significant effect of restricted feeding day (*F*_*13*,*455*_ = 67.9, *p* < 0.0001), but no main effect of mealtime (*F*_*1*,*35*_ = 0.001; *p* = 0.968). Day-fed mice weighed less than night fed mice on days 2, 4 and 5 of restricted feeding, but these differences were not significant after correction for multiple comparisons, and the trend was not sustained beyond day 5 (Supplementary Fig. S4B).

### Experiment 2. Daytime feeding shifts clock gene expression in the hippocampus

**Figure 3:**
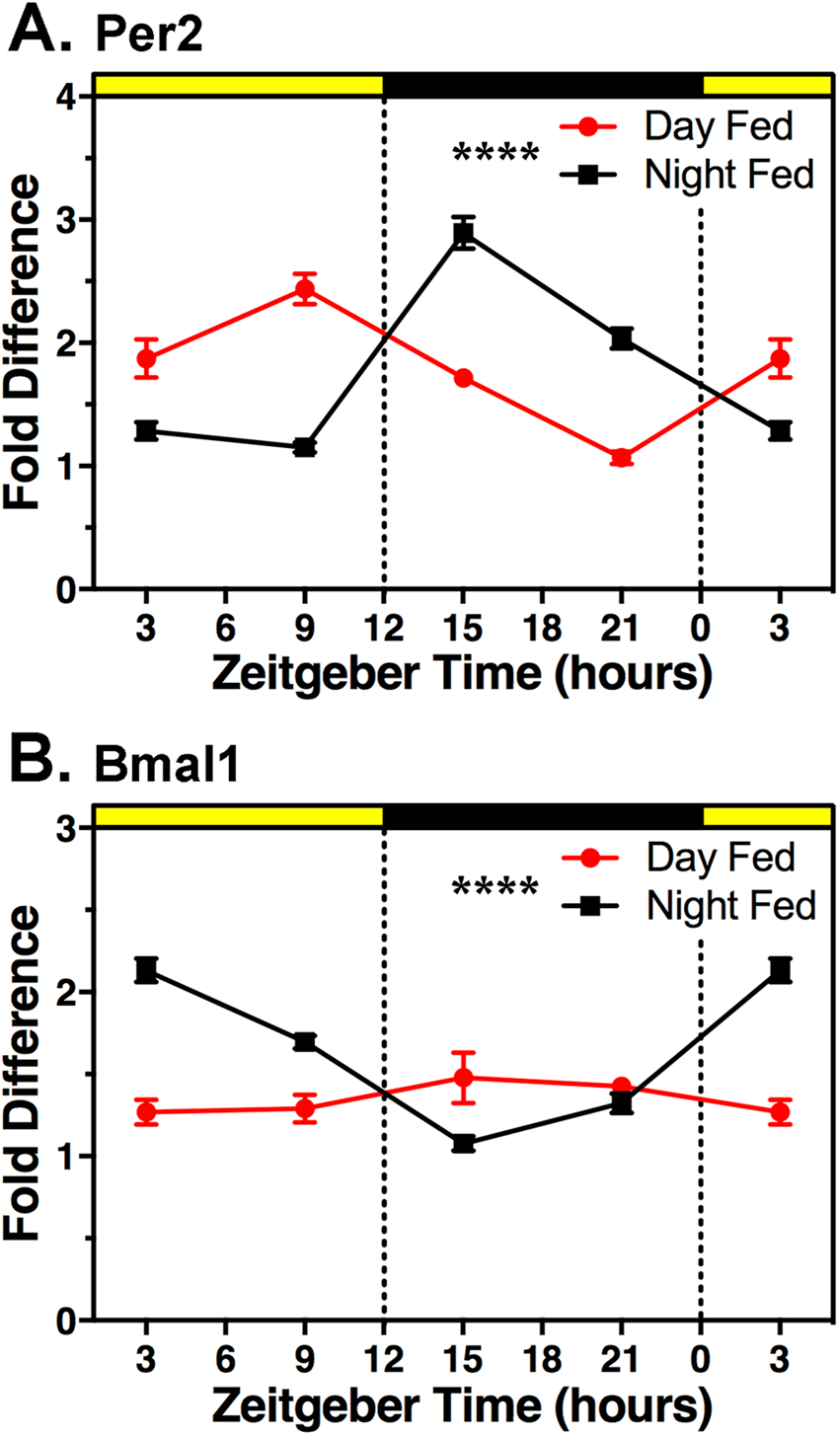
Effects of feeding schedules and time of day on clock gene expression in the hippocampus. A. *Per2*. B. *Bmal1*. Data are plotted as mean ± SEM (N = 3–5 mice per time point, per feeding condition; ZT3 is double-plotted to aid visualization). Red circles and lines are day-fed groups. Black squares and lines are night-fed groups. There was a significant main effect of time of day in both groups and a significant interaction between time of day and feeding condition (****p < 0.0001).

To confirm that circadian clock genes in the hippocampus of day-fed mice were shifted relative to night-fed mice, the dorsal hippocampus from mice in Experiment 2 was extracted at ZT3, 9, 15, or 21 for quantification of *Bmal1* and *Per2* expression using qPCR (Fig. 3). One-way ANOVA revealed a significant effect of time of day on *Bmal1* expression in both day-fed (*F*_*3*,*14*_ = 5.327, *p* = 0.012) and night-fed mice (*F*_*3*,*14*_ = 284.8, *p* < 0.0001), and on *Per2* expression in both day-fed (*F*_*3*,*14*_ = 111.9, *p* < 0.0001) and night-fed mice (*F*_*3*,*14*_ = 394.7, *p* < 0.0001). A two-way ANOVA revealed a significant interaction between feeding schedule and time of day for both *Per2* expression (*F*_*3*_,_28_ = 71.77, *p* < 0.0001) and *Bmal1* expression (*F*_3,28_ = 25.50, *p* < 0.0001). Day-fed and night-fed groups differed significantly at all 4 time points for both genes (*p* < . 05, Sidak’s multiple comparison test).

### Experiment 3a. Daytime feeding does not impair spontaneous alternation

A third cohort of day-fed and night-fed mice were tested for spontaneous alternation and contextual fear conditioning. After 14 days of restricted feeding, separate groups of day-fed and night-fed mice were place in a Y-maze for 8 min at either ZT2 (daytime) or ZT14 (nighttime) (Supplementary Fig. S1). There was a significant effect of mealtime on the total number of arm entries (*F*_1,36_ = 15.12, *p* = 0.0004), but no significant effect of test time (*F*_1,36_ = 2.98, *p* = 0.093) and no significant interaction (*F*_1,36_ = 0.44, *p* = 0.51). Night-fed mice exhibited more arm entries than day-fed mice at both test times (p < 0.05). However, the number of alternations as a percent of total possible alternations showed no effect of mealtime (*F*_1,36_ = 0.65, *p* = 0.43) or test time (*F*_1,36_ = 0.58, *p* = 0.45), and no significant interaction (*F*_1,36_ = 0.91, *p* = 0.34) (Fig. 1C).

### Experiment 3b. Daytime feeding does not impair contextual fear conditioning

On the second day after the spontaneous alternation test, day-fed and night-fed mice were placed in an operant chamber at either ZT2 or ZT14, and received two foot shocks separated by 64 sec. The mice were returned to the chamber 24 h later and freezing behavior was quantified (Supplementary Fig. S1). Day-fed and night-fed groups showed more freezing during day tests compared to night tests (*F*_*1*,*35*_ = 4.26, *p* = 0.046), but there was no effect of feeding schedule (*F*_*1*,*35*_ = 2.97, *p* = 0.093), and no significant interaction (*F*_*1*,*35*_ = 0.27, *p* = 0.61) (Fig. 1D).

## Discussion

The mammalian circadian system is comprised of a master light-entrainable pacemaker in the SCN, and many so-called peripheral or local oscillators in other brain regions, organs and tissue^27–29^. The SCN pacemaker mediates entrainment to LD cycles, and coordinates the timing of oscillators elsewhere via direct and indirect pathways^4,5,30,31^. Among the indirect pathways is SCN control of the daily rhythm of feeding, which induces a range of physiological responses (e.g., metabolic hormones) that participate in phase control of clocks in many tissues, with the SCN pacemaker being a notable exception^6–8^. The circadian system is designed to entrain to the solar day and accommodate gradual changes in day length with time of year^32,33^. The system is not designed for large, rapid shifts of the LD cycle, simulating jet travel or shift work rotations. Under these conditions, coupled oscillators within the SCN pacemaker can transiently dissociate, and clocks in other brain regions and organs may shift at different rates or in different directions, depending on intrinsic properties and the tissue-specific time cues to which they respond^34–40^. The result is transient internal desynchrony that resolves when re-entrainment is complete. If stable internal temporal order is important for optimal functioning of neural and physiological systems, then these functions may be impaired during desynchrony. This general prediction is supported by a variety of results, including evidence for impairment of hippocampus-dependent memory processes by LD cycle shifts^18–21,41^.

In the present study, we examined the functional consequences of a different form of internal ‘desynchrony’, one that is induced when feeding and LD cycles are in conflict. Nocturnal animals eat primarily at night, but if food is available only in the middle of the day, there is a marked realignment of circadian clocks throughout the brain and body. The net result is clearly adaptive, by ensuring that the organism is awake at the right time to find food, and prepared physiologically for a large influx of nutrients. In the presence of an LD cycle, the SCN pacemaker and a few other tissues (e.g., the pineal gland) are shifted relatively little or not at all by restricted daytime feeding, and thus there is substantial change in the phase relations between the minority of clocks that remain entrained to LD, and the majority, including the hippocampus, that align with mealtime. This form of internal ‘desynchrony’ differs from that induced by LD shifts because it is stable, beneficial for survival (certainly in the short term), and induced by conditions that may occur in natural habitats. It therefore would seem more appropriate to designate this as a state of ‘altered internal synchrony’ rather than ‘desynchrony’.

Given that the circadian response to a stable change in food availability is both coordinated and adaptive, the report that this was accompanied by impaired ability to remember objects and dangerous places was unexpected^12^. To explore this apparent paradox, we first evaluated the possibility that performance on these tests exhibits a circadian variation that is shifted by mealtime. In the study of Loh *et al*.^12^, NOR was assessed either late in the light or the dark period, in separate groups, and day-fed mice were impaired at both test times. We therefore tested mice at those times and two additional times of day early in the light and dark periods, when the mice would be active in anticipation of food availability. To increase statistical power, we tested a larger sample and used a repeated measures design, with unique sets of objects at each of the test times. We also maintained the restricted feeding schedules for a longer duration, to ensure that entrainment to mealtime was stable in day-fed and night-fed groups. With this procedure, we observed no impairment of object recognition in day-fed mice relative to night-fed mice, and no circadian variation in performance in either group. A similar absence of day-night differences in object recognition and spatial memory (Morris Water Maze test) in mice has been reported by others^42^.

As we had modified the procedures of Loh *et al*.^12^ in several ways, we next attempted a more formal replication, a necessary first step to identify variables that might underlie the different outcomes. With the assistance of the original authors, we recreated the apparatus, feeding protocols, and training and testing procedures used in their study, down to the brand of cleaner used in the chambers after each test. We again observed no deficit in object memory in the day-fed group compared to the night-fed group, and no evidence of circadian variation.

Performance on the NOR test as configured by Loh *et al*.^12^, and in the present study, requires perirhinal cortex but not the hippocampus^43,44^. We therefore assessed spontaneous alternation and contextual fear conditioning, two spatial memory assays known to be hippocampus-dependent, in a third cohort of mice. Day-fed and night-fed groups again showed equivalent performance on these tests. In the fear conditioning experiment, freezing scores were higher during the day tests compared to the night tests, a result consistent with several previous studies^21,45^.

There are many possible reasons why a particular result may fail to replicate between or even within labs^46^. Tests of cognitive function that rely on spontaneous behavior, such the NOR test, are particularly vulnerable to environmental conditions and the state of the animals. For example, external noise or smells may cause mice to be distracted and show a pattern of exploration that is not driven exclusively by an innate preference for novelty. Similarly, anxiety, hunger or sleepiness may affect exploration and limit the utility of the task for assessing memory. We were not able to reproduce the findings of Loh *et al*.^12^, and there may be subtle, unidentified methodological differences at play. Alternatively, there is the issue of statistical power and chance, which we attempted to address *a priori* by employing larger sample sizes and a within-subject design. Based on the present results, we conclude that daytime feeding schedules that induce a stable rhythm of food anticipation and that shift the hippocampus clock do not impair memory functions mediated by the hippocampus or perirhinal cortex. Stable reorganization of central and peripheral circadian clocks in response to daily feeding schedules may therefore be best construed as an adaptive re-alignment of the circadian system, rather than a maladaptive misalignment.

## Electronic supplementary material

Supplementary table1 and figures1–4

## Data Availability

The datasets analysed in the current study are available from the corresponding author on request.
