# Supplementary material for: Midday meals do not impair mouse memory
Source: Sci Rep. 2018 Nov 19;8:17013. doi: 10.1038/s41598-018-35427-y (PMC6242856; doi:10.1038/s41598-018-35427-y)

Supplementary Materials

Midday meals do not impair mouse memory

Sarah C. Power, Mateusz J. Michalik, Sylvie Couture-Nowak,

Brianne A. Kent, Ralph E. Mistlberger

Table S1. Primers used for qPCR

Gene	Forward	Reverse
Per2	*ACCTCCCTGCAGACAAGAA*	*CTCATTAGCCTTCACCTGCTT*
Bmal1	*GTGCCACTGACTACCAAGAAAG*	*CCCTTGCATTCTTGATCCTTCC*
Rplp0	*GAGAAACTGCTGCCTCACATC*	*CAGCAGCTGGCACCTTATT*

Supplementary figures

Figure S1. Experiment apparatus and timelines (for day-fed groups). Green shading denotes mealtimes. 'RF' denotes restricted feeding day. 'S' denotes familiarization trials, and 'C' denotes test trials with one familiar object, and one novel object. 'H' denotes habituation to the test apparatus.

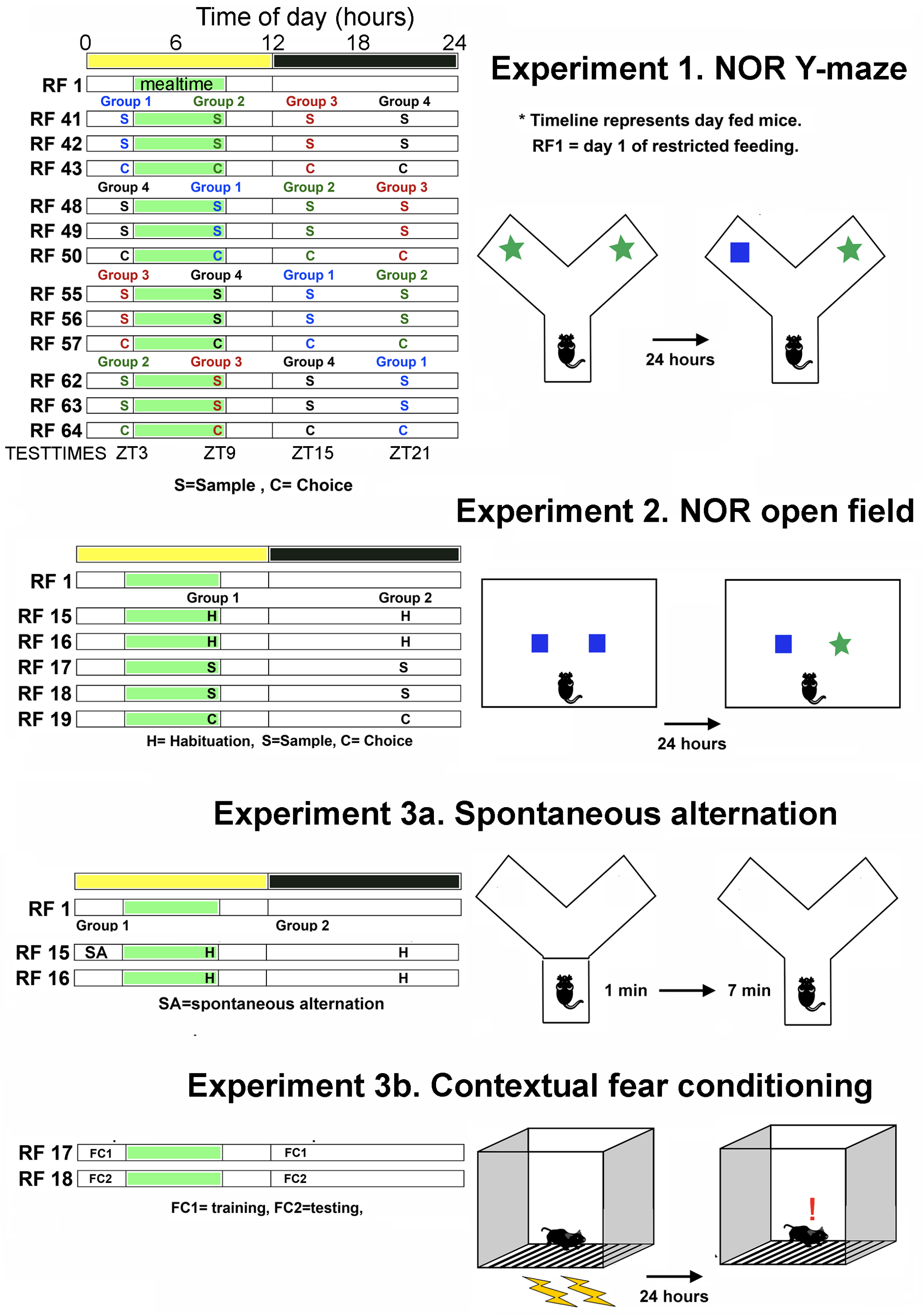

Figure S2. Discrimination index ratios for individual mice at each test time in Experiment 1.

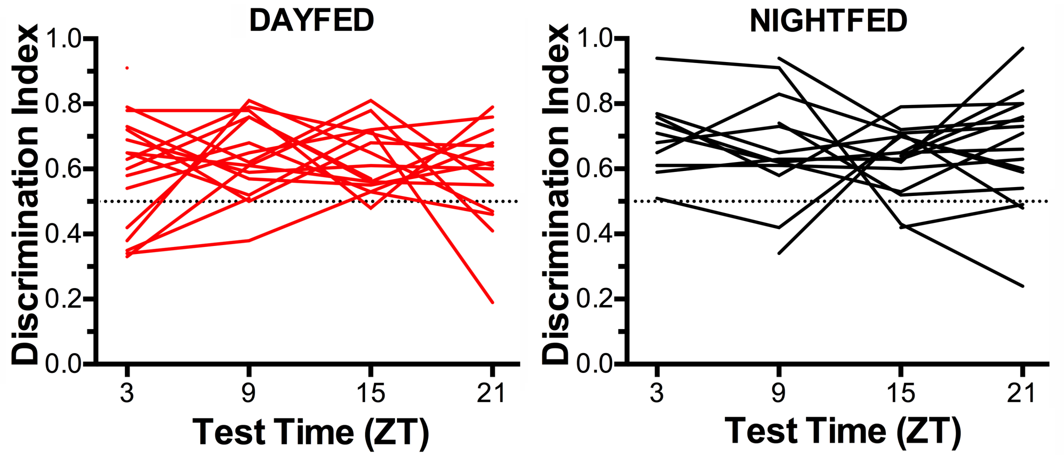

Figure S3. Total exploration time (both objects) in day-fed mice (red lines and circles) and night-fed mice (black lines and squares) in Experiments 1 and 2.

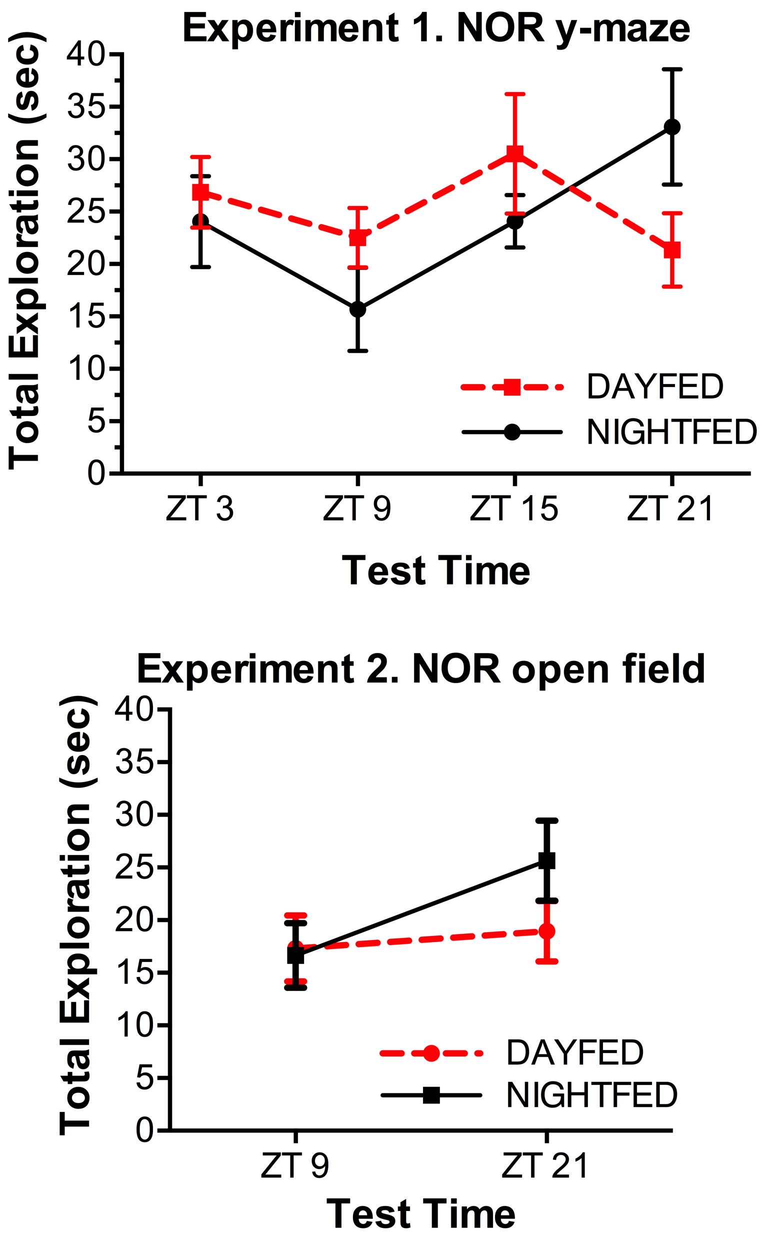

Figure S4. Group mean (± SEM) food intake (A) and body weight (B) during the first 14 days of restricted feeding in Experiment 2. Day-fed mice (red lines and circles) ate less than night-fed mice (black lines and squares) on days 1, 3 and 4 (*p<.05 between groups, corrected for multiple tests).

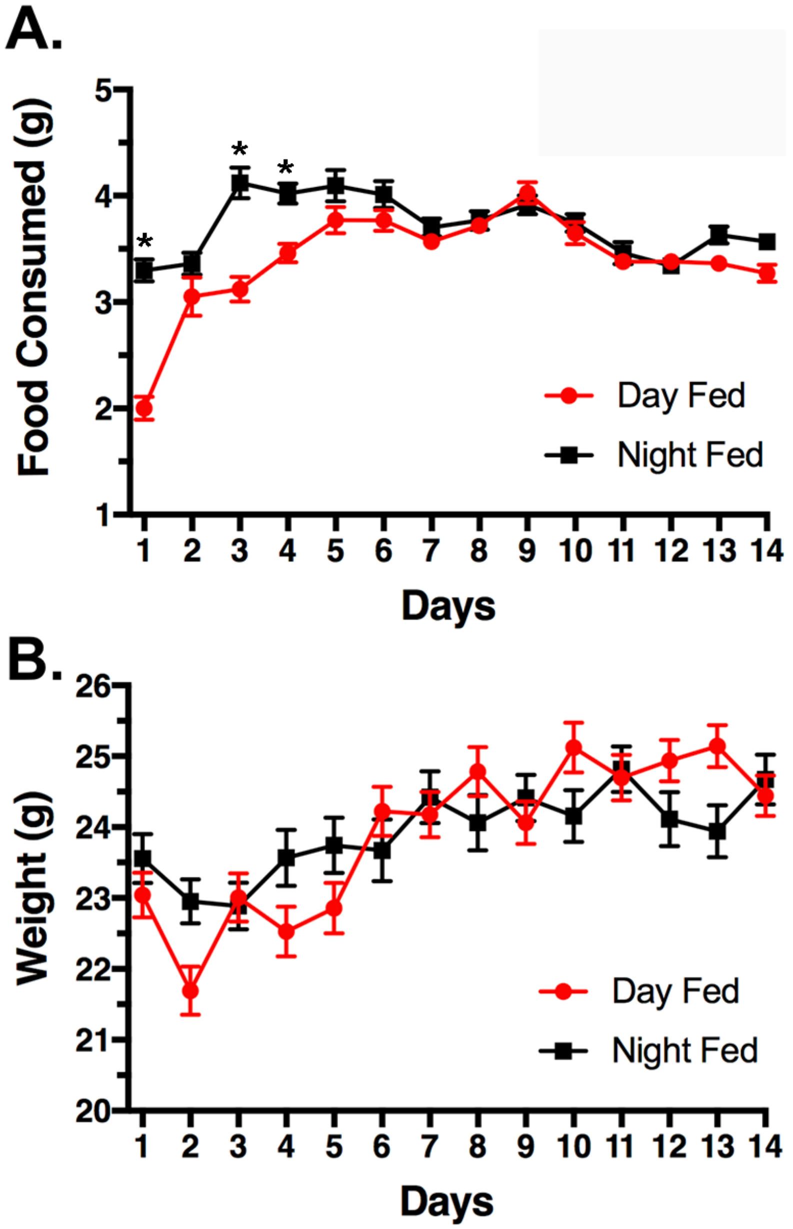

Supplement: Supplementary file 1 — Supplementary table1 and figures1–4 [file 41598_2018_35427_MOESM1_ESM.pdf]
